# Supplementary material for: Multi‐dataset identification of innovative feature genes and molecular mechanisms in keratoconus
Source: J Cell Mol Med. 2024 Sep 19;28(18):e70079. doi: 10.1111/jcmm.70079 (PMC11412914; doi:10.1111/jcmm.70079)
Supplement: Supplementary file 2 — Material S2. [file JCMM-28-e70079-s002.zip › 7.GSEA analysis/6.SNORA74B/KEGG_STEROID_HORMONE_BIOSYNTHESIS.html]

Details for gene set KEGG\_STEROID\_HORMONE\_BIOSYNTHESIS[GSEA]

|  || Dataset | SNORA74B.group.cls#h\_versus\_l |
| Phenotype | group.cls#h\_versus\_l |
| Upregulated in class | l |
| GeneSet | KEGG\_STEROID\_HORMONE\_BIOSYNTHESIS |
| Enrichment Score (ES) | -0.4447191 |
| Normalized Enrichment Score (NES) | -1.3608408 |
| Nominal p-value | 0.04288499 |
| FDR q-value | 0.3386223 |
| FWER p-Value | 0.925 |
Table: GSEA Results Summary

  

Fig 1: Enrichment plot: KEGG\_STEROID\_HORMONE\_BIOSYNTHESIS      
 Profile of the Running ES Score & Positions of GeneSet Members on the Rank Ordered List

  

| SYMBOL | TITLE | RANK IN GENE LIST | RANK METRIC SCORE | RUNNING ES | CORE ENRICHMENT || 1 | UGT1A8 | na | 321 | 0.680 | 0.0577 | No |
| 2 | UGT1A6 | na | 2066 | 0.366 | 0.0434 | No |
| 3 | CYP7A1 | na | 3254 | 0.288 | 0.0376 | No |
| 4 | UGT2B4 | na | 3661 | 0.272 | 0.0526 | No |
| 5 | HSD3B2 | na | 4459 | 0.244 | 0.0537 | No |
| 6 | CYP17A1 | na | 4721 | 0.235 | 0.0693 | No |
| 7 | SULT2B1 | na | 5486 | 0.209 | 0.0678 | No |
| 8 | UGT2B11 | na | 6088 | 0.190 | 0.0692 | No |
| 9 | CYP11B2 | na | 7463 | 0.149 | 0.0443 | No |
| 10 | UGT2B28 | na | 7492 | 0.149 | 0.0581 | No |
| 11 | CYP3A4 | na | 8000 | 0.136 | 0.0569 | No |
| 12 | HSD17B12 | na | 8533 | 0.124 | 0.0537 | No |
| 13 | HSD3B1 | na | 9524 | 0.102 | 0.0353 | No |
| 14 | UGT2B10 | na | 9771 | 0.097 | 0.0377 | No |
| 15 | SRD5A1 | na | 10017 | 0.092 | 0.0397 | No |
| 16 | HSD17B1 | na | 13258 | 0.044 | -0.0494 | No |
| 17 | UGT2A3 | na | 13387 | 0.043 | -0.0488 | No |
| 18 | CYP11B1 | na | 13859 | 0.038 | -0.0587 | No |
| 19 | HSD17B8 | na | 15787 | 0.017 | -0.1126 | No |
| 20 | CYP11A1 | na | 17321 | 0.001 | -0.1567 | No |
| 21 | COMT | na | 18119 | -0.007 | -0.1789 | No |
| 22 | SRD5A2 | na | 18577 | -0.013 | -0.1909 | No |
| 23 | STS | na | 20542 | -0.035 | -0.2441 | No |
| 24 | HSD17B7 | na | 20743 | -0.037 | -0.2462 | No |
| 25 | UGT2A1 | na | 21014 | -0.040 | -0.2501 | No |
| 26 | CYP3A7 | na | 21996 | -0.052 | -0.2733 | No |
| 27 | UGT2B15 | na | 25363 | -0.105 | -0.3600 | No |
| 28 | SRD5A3 | na | 26842 | -0.136 | -0.3892 | No |
| 29 | CYP21A2 | na | 26928 | -0.138 | -0.3781 | No |
| 30 | AKR1D1 | na | 28010 | -0.165 | -0.3930 | No |
| 31 | CYP7B1 | na | 28874 | -0.191 | -0.3991 | No |
| 32 | CYP3A43 | na | 30456 | -0.243 | -0.4208 | Yes |
| 33 | AKR1C1 | na | 30669 | -0.251 | -0.4021 | Yes |
| 34 | UGT2B7 | na | 31877 | -0.316 | -0.4058 | Yes |
| 35 | HSD11B2 | na | 31945 | -0.320 | -0.3762 | Yes |
| 36 | HSD17B3 | na | 32195 | -0.334 | -0.3505 | Yes |
| 37 | CYP3A5 | na | 32259 | -0.338 | -0.3191 | Yes |
| 38 | HSD17B2 | na | 32695 | -0.367 | -0.2955 | Yes |
| 39 | HSD11B1 | na | 32795 | -0.376 | -0.2613 | Yes |
| 40 | AKR1C3 | na | 32884 | -0.384 | -0.2261 | Yes |
| 41 | AKR1C4 | na | 33005 | -0.394 | -0.1907 | Yes |
| 42 | SULT1E1 | na | 33365 | -0.432 | -0.1585 | Yes |
| 43 | CYP19A1 | na | 33383 | -0.435 | -0.1162 | Yes |
| 44 | CYP1A1 | na | 33610 | -0.466 | -0.0768 | Yes |
| 45 | HSD17B6 | na | 33889 | -0.510 | -0.0346 | Yes |
| 46 | CYP1B1 | na | 34253 | -0.597 | 0.0137 | Yes |
Table: GSEA details [plain text format]

  

Fig 2: KEGG\_STEROID\_HORMONE\_BIOSYNTHESIS      
 Blue-Pink O' Gram in the Space of the Analyzed GeneSet

  

Fig 3: KEGG\_STEROID\_HORMONE\_BIOSYNTHESIS: Random ES distribution      
 Gene set null distribution of ES for **KEGG\_STEROID\_HORMONE\_BIOSYNTHESIS**
